# Tgf-β1 Inhibits Cftr Biogenesis and Prevents Functional Rescue of ΔF508-Cftr in Primary Differentiated Human Bronchial Epithelial Cells

**DOI:** 10.1371/journal.pone.0063167

**Published:** 2013-05-09

**Authors:** Steven M. Snodgrass, Kristine M. Cihil, Pamela K. Cornuet, Michael M. Myerburg, Agnieszka Swiatecka-Urban

**Affiliations:** 1 Division of Pediatric Pulmonology, Children’s Hospital of Pittsburgh, Pittsburgh, Pennsylvania, United States of America; 2 Department of Cell Biology and Physiology, University of Pittsburgh School of Medicine, Pittsburgh, Pennsylvania, United States of America; 3 Division of Pulmonary, Allergy and Critical Care Medicine, University of Pittsburgh School of Medicine, Pittsburgh, Pennsylvania, United States of America; 4 Division of Pediatric Nephrology, Children’s Hospital of Pittsburgh, Pittsburgh, Pennsylvania, United States of America; University of Tübingen, Germany

## Abstract

CFTR is an integral transmembrane glycoprotein and a cAMP-activated Cl^−^ channel. Mutations in the *CFTR* gene lead to Cystic Fibrosis (CF)–an autosomal recessive disease with majority of the morbidity and mortality resulting from airway infection, inflammation, and fibrosis. The most common disease-associated mutation in the *CFTR* gene–deletion of Phe508 (ΔF508) leads to a biosynthetic processing defect of CFTR. Correction of the defect and delivery of ΔF508-CFTR to the cell surface has been highly anticipated as a disease modifying therapy. Compared to promising results in cultured cell this approach was much less effective in CF patients in an early clinical trial. Although the cause of failure to rescue ΔF508-CFTR in the clinical trial has not been determined, presence of factor(s) that interfere with the rescue *in vivo* could be considered. The cytokine TGF-β1 is frequently elevated in CF patients. TGF-β1 has pleiotropic effects in different disease models and genetic backgrounds and little is known about TGF-β1 effects on CFTR in human airway epithelial cells. Moreover, there are no published studies examining TGF-β1 effects on the functional rescue of ΔF508-CFTR. Here we found that TGF-β1 inhibits CFTR biogenesis by reducing mRNA levels and protein abundance in primary differentiated human bronchial epithelial (HBE) cells from non-CF individuals. TGF-β1 inhibits CFTR biogenesis without compromising the epithelial phenotype or integrity of HBE cells. TGF-β1 also inhibits biogenesis and impairs the functional rescue of ΔF508-CFTR in HBE cells from patients homozygous for the ΔF508 mutation. Our data indicate that activation of TGF-β1 signaling may inhibit CFTR function in non-CF individuals and may interfere with therapies directed at correcting the processing defect of ΔF508-CFTR in CF patients.

## Introduction

The cystic fibrosis transmembrane conductance regulator (CFTR) is an integral transmembrane glycoprotein from the family of ATP binding cassette (ABC) transporters. CFTR forms a cAMP-activated Cl^−^ channel that mediates transepithelial Cl^−^ secretion in various fluid-transporting epithelia [1]–[3]. In the airway, CFTR plays a critical role in regulating mucociliary clearance by maintaining airway surface liquid [4], [5].

Mutations in the *CFTR* gene lead to Cystic Fibrosis (CF)–the most common fatal genetic disorder in Caucasians. CF pathophysiology centers on the defective function of CFTR in various tissues, most prominently the exocrine pancreas and airway. The most common disease-associated mutation in the *CFTR* gene–deletion of Phe508 (ΔF508) leads to a temperature sensitive processing defect of the ΔF508-CFTR protein. ΔF508-CFTR is retained in the endoplasmic reticulum (ER) in an immature, partially glycosylated form [6]. Low temperature and chemical chaperones rescue the biosynthetic processing defect and allow exit of ΔF508-CFTR from the ER, maturation while passing through the Golgi complex, and trafficking to the cell membrane. Because rescued ΔF508-CFTR is partially functional as a Cl^−^ channel, correction of the processing defect to deliver ΔF508-CFTR to the cell surface has been highly anticipated as a disease modifying therapy [7], [8]. Several small molecules targeting defective biosynthetic processing of ΔF508-CFTR, called CFTR correctors have been identified [9]. The only corrector that reached a clinical trial, VX-809 failed to mature ΔF508-CFTR, did not rescue the functional defect of ΔF508-CFTR in nasal epithelium and did not improve lung function in patients homozygous for ΔF508 mutation despite partially correcting ΔF508-CFTR in cultured cells [7], [10]. Failure of VX-809 to rescue the ΔF508-CFTR defect in CF patients raises suspicion for presence of factor(s) that interfere with the ΔF508-CFTR rescue *in vivo*.

CF patients are born with a structurally normal airway but over the years acquire chronic infections and develop inflammation, remodeling and fibrosis [11]–[13]. It is unknown how the pro-inflammatory and pro-fibrotic CF environment may affect therapies directed at correcting the processing defect of ΔF508-CFTR. Studies demonstrate that cytokines such as interferon gamma and several interleukins including interleukin 1ß, 4, and 13 regulate CFTR in different cellular models [14]–[18]. TGF-β1 is the major plasma isoform of TGF-β–an extracellular cytokine with immunomodulatory and pro-fibrotic properties [19]. TGF-β1 plays a key role in the pathophysiology of a number of pulmonary disorders, including CF [20]–[23]. TGF-β1 signaling occurs through the cell membrane associated transmembrane serine/threonine kinase receptor (TβR) I and II [24]. TGF-β1 induces assembly of TβRI and TβRII into a complex at the cell surface followed by phosphorylation of Smad transcription factors, nuclear translocation of activated Smads, and transcriptional responses [25].

Nothing is known about the effects of TGF-β1 signaling on CFTR in primary differentiated human bronchial epithelial cells. In other cell models TGF-β1 was shown to interfere with CFTR expression and function [26]–[28]. Because TGF-β1 signaling demonstrates tissue-specific and cell culture-dependent effects [29], [30] it is practically impossible to extrapolate results from other models, including cells heterologously expressing CFTR to predict the effects of TGF-β1 on endogenous CFTR in primary differentiated human bronchial epithelial cells. Moreover, there are no published data examining effects of TGF-β1 on ΔF508-CFTR.

Studies conducted in primary differentiated human bronchial epithelial cells from non-CF individuals (HBE) and from CF patients homozygous for the ΔF508 mutation (CF-HBE) show that TGF-β1 inhibits CFTR biogenesis by reducing CFTR mRNA levels and protein abundance. Moreover, TGF-β1 inhibits biogenesis of ΔF508-CFTR and interferes with the corrector mediated functional rescue of ΔF508-CFTR.

## Materials and Methods

### Cell Lines and Cell Culture

Primary differentiated human bronchial epithelial cells (HBE cells; homozygous WT-CFTR and CF-HBE; homozygous ΔF508-CFTR) were received from Dr. Joseph Pilewski (Cystic Fibrosis Research Center Epithelial Cell Core at the University of Pittsburgh School of Medicine, Pittsburgh, PA) as previously described [31]–[33]. Cells were cultured on human placental collagen-coated Costar Transwell filters (0.33 cm^2^ at density of ∼ 2×10^5^/cm^2^ or 1.12 cm^2^ at density ∼ 7×10^5^/cm^2^ as previously described and used for experimentation following 6–8 weeks of culture at an air-liquid interface [34], [35].

*Antibodies and Reagents*–The following antibodies were used: anti-human CFTR (596; Cystic Fibrosis Foundation Therapeutics, Inc.; Chapel Hill, NC), anti-ezrin, anti-E-cadherin, anti-N-cadherin (BD Biosciences, San Jose, CA), anti-Smad2 (Clontech, Mountain View, CA), anti-phospho-Smad2, anti-TβRI, anti-Na,K-ATPase (Millipore; Billerica, MA), anti-Lamin B1 (Abcam, Cambridge, MA), and horseradish peroxide-conjugated secondary antibodies (BioRad Laboratories; Hercules, CA). All antibodies were used at the concentrations recommended by the manufacturer. Human TGF-β1 (Sigma, St. Louis, MO), the TβRI inhibitor SB431542 (Stemgent, San Diego, CA), Complete Protease Inhibitor Cocktail and PhosSTOP phosphatase inhibitor cocktail tablets (Roche Applied Science, Indianapolis, IN).

### Real-time Quantitative Reverse-Transcription (qRT)-PCR

Total RNA was isolated on two separate occasions from triplicate individual cultures of different HBE and CF-HBE cells per condition using the RNeasy mini kit (Qiagen, Valencia, CA) according to the manufacturer’s instructions, with additional on-column DNase treatment with the RNase-free DNase Set (Qiagen) to remove contaminating genomic DNA for downstream applications. Real time reactions were run in triplicates with each reaction emanating from a starting sample amount of 20 ng total RNA before Reverse Transcription to cDNA. Superscript II Reverse Transcriptase (Invitrogen, Grand Island, NY) was used to generate cDNA from total RNA. qRT-PCR was performed using ABsolute™ Blue QPCR SYBR® Green ROX Mix (Thermo Scientific, Walthman, MA) and ABI PRISM® 7900HT Sequence Detection System (Applied Biosystems, Foster City, CA) according to the manufacturer’s instructions. The primer sequences for CFTR RNA were from the Harvard Medical School Primer Bank (ID#09421312c2; CFTR-213 forward: 5′- TGCCCTTCGGCGATGTTTTT -3′ and CFTR-339 reverse: 5′- GTTATCCGGGTCATAGGAAGCTA -3′) [36]. The primer sequences for GAPDH RNA were self-selected (forward: 5′-TGACAACTTTGGTATCGTGGAAGG-3′ and reverse: 5′-AGGGATGATGTTCTGGAGAGCC-3′). All reactions were performed in triplicates. Fluorescence emission was detected for each PCR cycle, and the threshold cycle (Ct) values and the average Ct of the triplicate reactions were determined for CFTR and GAPDH. The Ct value was defined as the actual PCR cycle when the fluorescence signal increased above the background threshold, and the ΔCt was determined for each sample by subtracting the Ct for GAPDH from the Ct for CFTR, and the mean ΔCt of the triplicate samples was determined. The ΔΔCt was calculated by subtracting the ΔCt for the negative control (CTRL) treated cells from the ΔCt for the TGF-ß1 treated cells at different time points. Fold change values were determined according to the following formula: 2^−ΔΔCT^.

### Isolation of Nuclear Fractions

Isolation of nuclear fractions in HBE cells to detect the nuclear translocation of activated Smad2 after TGF-ß1 treatment was carried out with the Nuclear Protein Isolation-Translocation Kit (FIVEphoton Biochemicals, San Diego, CA) according to the manufacturer’s instructions.

### Biochemical Determination of Plasma Membrane Proteins and Western Blotting

The biochemical determination of proteins in the apical or basolateral plasma membrane was performed by domain selective plasma membrane biotinylation as described previously [37]. Permeable growth supports containing cells grown in air-liquid interface were transferred quickly from the cell culture incubator to a 6-well plate filled with phosphate buffered saline containing 1 mM MgCl_2_ and 0.1 mM CaCl_2_ (PBS++) at 4°C to stop membrane trafficking. PBS++ was suctioned and the cell membrane impermeable EZ-Link™ Sulfo-NHS-LC-Biotin (Pierce Chemicals, Co., Rockford, IL; 0.8 mg/ml of PBS++) was added for 30 minutes at 4°C to the apical or basolateral side of growth supports to biotinylate the apical or basolateral membrane proteins, respectively. Following a thorough washing with PBS++ cells were lysed in buffer containing 25 mM HEPES, pH 8.0, 1% Triton, 10% glycerol, 1 mM Na3VO4, and Complete Protease Inhibitor Cocktail (Roche Applied Science, Indianapolis, IN). Cell lysates were centrifuged at 14,000 g for 10 minutes at 4°C and 10% of the supernatants was mixed 1∶2 with Laemli buffer (BioRad Laboratories, Inc., Hercules, CA) containing 100 mM DTT at 37°C to prepare whole cell lysates (WCL). Biotinylated proteins were isolated from the remaining supernatants with streptavidin agarose and eluted into Laemli buffer containing 100 mM DTT at 85°C. CFTR, TβRI, and Na,K-ATPase were visualized by Western blotting with appropriate primary and secondary horseradish peroxidase antibodies using the Western Lightning™ Plus-ECL detection system (Perkin Elmer Inc.; Waltham, MA) followed by chemiluminesence. Protein abundance was quantified by densitometry using exposures within the linear dynamic range of the film [37].

### Short-Circuit Recordings

The short circuit currents (*I_SC_*) were measured in Ussing-type chambers (Physiological Instruments; San Diego, CA) as previously described [34]. In brief, primary HBE cells cultured on filter supports were mounted in modified Ussing chambers (P2300, Physiological Instruments) with custom sliders modified to fit the Transwell inserts, and the cultures were continuously short circuited with an automatic voltage clamp (Department of Bioengineering, University of Iowa, Iowa City, IA). Transepithelial resistance was measured by periodically applying a 2.5-mV bipolar voltage pulse and was calculated using Ohm’s law. The bathing Ringer’s solution was composed of 120 mM NaCl, 25 mM NaHCO_3_, 3.3 mM KH_2_PO_4_, 0.8 mM K_2_HPO_4_, 1.2 mM MgCl_2_, 1.2 mM CaCl_2_, and 10 mM glucose. Chambers were constantly gassed with a mixture of 95% O2 and 5% CO2 at 37°C, which maintained the pH at 7.4. Following an equilibration period, the baseline *I_SC_* was recorded. Amiloride (10 µM) was added to the apical bath solution to inhibit Na^+^ absorption through ENaC. Subsequently, *Isc* was stimulated with the cAMP agonist, forskolin (10 µM) added to the apical and basolateral bath solutions followed by thiazolidonone CFTR inhibitor CFTR_inh_-172 (20 µM) added to the apical bath solution to inhibit CFTR-mediated *Isc.* Data are expressed as the CFTR_inh_-172 sensitive *I*sc calculated by subtracting the *Isc* after CFTR_inh_-172 treatment from the peak forskolin-stimulated *Isc*.

### Rescue of Cell Surface Delivery of ΔF508-CFTR

To increase delivery of ΔF508-CFTR to the apical membrane we used CFTR corrector VX-809 (Selleckchem, Houston, TX) [7]. Additional experiments were performed with corrector CF-106951 (also known as VRT-534; Cystic Fibrosis Therapeutics, Inc., Bethesda, MD) [38]. VX-809 (10 µM), CF-106951 (10 µM) or vehicle control (DMSO) was added daily for 48 h to the basolateral medium of CF-HBE cells. The final concentration of DMSO was <0.1%.

### Data Analysis and Statistics

Statistical analysis of the data was performed using GraphPad Prism version 5.0 for Mac OS×(GraphPad Software Inc., San Diego, CA). The means were compared by a two-tailed t-test. A P value <0.05 was considered significant. Data are expressed as mean±standard error of the mean (S.E.M.).

## Results

### TβRI/II Polarizes to the Basolateral Membrane Domain in HBE Cells

**Figure 1:**
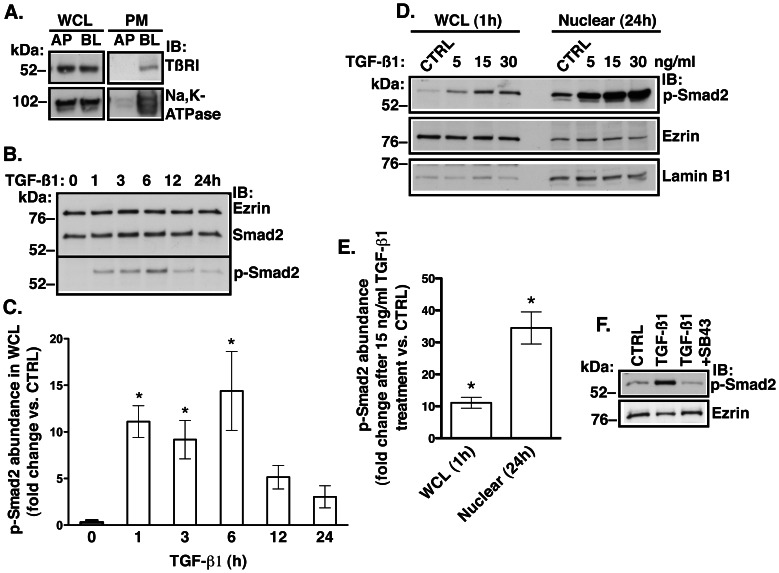
Western blot experiments demonstrating that TGF-β1 signals via the canonical, Smad2 pathway in HBE cells. (**A**) Representative experiment demonstrating polarization of a 6-week culture of HBE cells determined by the basolateral (BL) distribution of Na,K-ATPase. TβRI was detected in the BL membrane domain. The apical (AP) or BL plasma membrane (PM) proteins were isolated by selective cell surface biotinylation using cell membrane impermeable EZ-Link^a^ Sulfo-NHS-LC-Biotin. Protein abundance in whole cell lysate (WCL) did not differ. Experiment was repeated 3 times in cells from different donors with similar results. Representative experiment (**B**) and summary of data (**C**) demonstrating that abundance of phosphorylated Smad2 (p-Smad2) increased sharply in WCL after one hour of TGF-β1 (15 ng/ml) treatment and declined after 6 h. p-Smad2 was detected in WCL with anti-p-Smad2 specific antibody and normalized to the total Smad2. Ezrin was used as a loading control. The p-Smad2 abundance at different time points after TGF-β1 treatment was compared to the amount before treatment (time “0″). Representative experiment (**D**) and summary of data (**E**) demonstrating the nuclear translocation of p-Smad2 after TGF-β1 treatment for 24 h. Vehicle control (CTRL) or TGF-β1 (5, 15, or 30 ng/ml) was added to the basolateral medium in parallel experiments. Cells were lysed after either one hour of TGF-β1 treatment to confirm Smad2 phosphorylation in WCL or after 24 h treatment to examine the nuclear content of p-Smad2. Enrichment of lamin B1 and depletion of ezrin in the nuclear fraction confirms successful isolation of nuclear fraction. A 10-fold increase in p-Smad2 in WCL (similar to C) demonstrated activation of Smad2. A 30-fold increase in p-Smad2 in the nuclear fraction after 24 h of TGF-β1 treatment coinciding with the decline of p-Smad2 in WCL (B&C) indicates that p-Smad2 is translocated to the nucleus. (**B–E**) Experiments were repeated at least three times in HBE cells from different donors. *, *p*<0.05 *vs.* CTRL. Error bars, S.E.M. (**F**) The competitive TβRI inhibitor, SB431542 prevented TGF-β1 mediated increase in p-Smad2 abundance in WCL. Cells were incubated for one hour with vehicle control (CTRL), or TGF-β1 (15 ng/ml) in the absence or presence of SB431542 (1 µM). Ezrin was used as a loading control. Experiment was repeated 3 times in HBE cells from different donors with similar results.

The TβRI/TβRII complex is located at the plasma membrane [25]; however, little is known about membrane distribution of TβRI/TβRII in polarized or differentiated epithelial cells. Polarized distribution of TβRI/II has only been shown in canine kidney (MDCK) cells, where the TβRI/II complex was found in the basolateral membrane [39]. Because the TGF-β1 signaling varies greatly in different cell types and under different cell culture conditions [29], [30] we first examined localization of the TβRI/II complex in HBE cells. HBE cells–pass one were cultured for 6–8 weeks in air-liquid interphase to establish polarized monolayers. Localization of the TβRI/II complex was examined by selective cell surface biotinylation. The TβRI localized at the basolateral membrane (Fig. 1A). Similar distribution was observed for TβRII (data not shown).

### TGF-β1 Signaling is Activated by Clinically Relevant Concentrations of TGF-β1 in HBE Cells

There are no published studies examining activation of TGF-β pathway in HBE cells. Acting via the canonical pathway, TGF-β1 activates (i.e. phosphorylates) Smad2 transcription factor and induces nuclear translocation of activated Smad2 [25]. Clinically relevant concentration of TGF-β1–15 ng/ml [40] added to the basolateral medium of HBE monolayers induced phosphorylation of Smad2 in whole cell lysates (WCL) in a time dependent manner (Fig. 1B&C). The abundance of phosphorylated Smad2 (p-Smad2) decreased in WCL after the 6 h time point because p-Smad2 was translocated to the nucleus (Fig. 1D&E). The nuclear translocation of p-Smad2 was elicited by a range of TGF-β1 concentrations (Fig. 1D). The effect of TGF-β1 was specifically mediated by activating TβRI because the competitive inhibitor of TβRI, SB431542 blocked the TGF-β1 mediated effect and prevented Smad2 phosphorylation (Fig. 1F). These data demonstrate that at clinically relevant concentrations TGF-β1 activates the canonical TGF-β pathway in HBE cells.

### TGF-β1 Inhibits CFTR Biogenesis in HBE Cells

**Figure 2:**
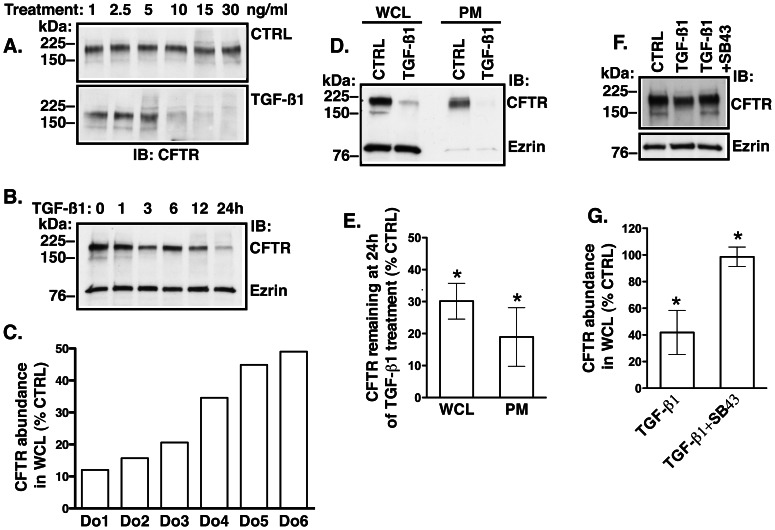
Western blot experiments demonstrating that TGF-β1 decreases CFTR protein abundance in HBE cells. (**A**) Representative experiment demonstrating that TGF-β1 decreased CFTR abundance in whole cell lysate (WCL) in a concentration-dependent manner. Different concentrations of TGF-β1 or vehicle control (CTRL) were added to the basolateral medium and cells were incubated for 24 h. CFTR was detected with anti-CFTR antibody CFF596. (**B**) Representative experiment demonstrating that TGF-β1 decreases WCL CFTR in a time-dependent manner. TGF-β1 (15 ng/ml) or vehicle control (CTRL) was added to the basolateral medium and cells were incubated for 24 h. Ezrin was used as a loading control. (**A&B**) Experiments were repeated three times in cells from different donors with similar results. (**C**) Representative experiment demonstrating effects of the 24 h treatment with TGF-β1 (15 ng/ml) on CFTR in WCL from six donors (Do). The underlying diagnoses were: Do#1, 4, & 6– no known lung disease; Do#2– interstitial pulmonary fibrosis; Do#3– emphysema; Do#5– sarcoidosis. Each bar represents two experiments. Representative experiment (**D**) and summary of data (**E**) demonstrating the effects of the 24 h treatment with TGF-β1 (15 ng/ml) on CFTR abundance in WCL and in the plasma membrane (PM). The apical plasma membrane proteins were isolated by selective plasma membrane biotinylation. Ezrin was used as a loading control. The absence of ezrin in the biotinylated samples confirms integrity of the HBE monolayers. 12 experiments in cells from 6 donors/group in WCL and 6 experiments from 6 donors/group in PM. *, *p*<0.05 *vs.* CTRL. Error bars, S.E.M.M. Representative experiment (**F**) and summary of data (**G**) demonstrating that the competitive TβRI inhibitor, SB431542 prevented the TGF-β1 mediated inhibition of CFTR abundance in WCL. Cells were incubated for 24 h with CTRL or TGF-β1 (15 ng/ml) in the absence or presence of SB431542. Ezrin was used as a loading control. 3 experiments in cell from different donors/group. *, *p*<0.05 *vs.* CTRL. Error bars, S.E.M.

Transcriptional responses elicited by TGF-β1 signaling affect gene expression and result in altered protein abundance [25]. We first examined the effects of TGF-β1 on CFTR protein. TGF-β1 was added to the basolateral medium at increasing concentrations for 24 h because by that time activated Smad2 had translocated to the nucleus to elicit transcriptional responses. TGF-β1 at concentrations similar to those that activated the canonical TGF-β pathway reduced the WCL abundance of CFTR (Fig. 2A *versus* Fig. 1D) and the most profound effect was observed at 24 h (Fig. 2B). We examined TGF-β1 effects on CFTR in HBE cells from different donors. TGF-β1 inhibited CFTR abundance by at least 50% in cells from all donors but the range of inhibition differed widely between cells from different donors (Fig. 2C). Depletion of the WCL CFTR was accompanied by its depletion from the plasma membrane (Fig. 2D&E). These effects were specifically mediated by TβRI because SB431542 blocked the TGF-β1 mediated CFTR depletion (Fig. 2F&G).

**Figure 3:**
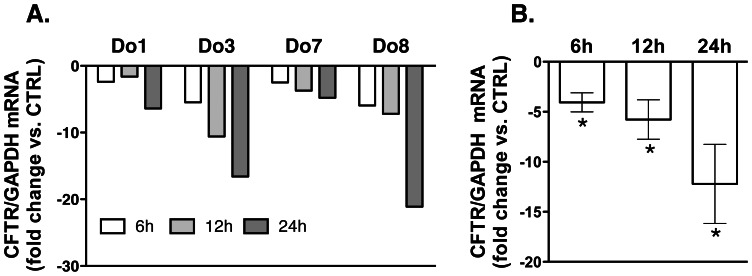
Real-time Quantitative Reverse-Transcription (qRT-PCR) experiments demonstrating that TGF-β1 decreases CFTR mRNA levels in HBE cells. TGF-β1 (15 ng/ml) or vehicle control (CTRL) was added to the basolateral medium and cells were incubated for 6, 12, or 24 h. Raw data were analyzed using the ΔΔC_t_ method. Changes in the CFTR mRNA were normalized to GAPDH. Data are expressed as fold change in CFTR mRNA *vs.* CTRL. All experiments were performed twice in triplicates in cell obtained from 4 donors (Do). Data from individual donors (A) and summary of Data (B). The underlying diagnoses were: Do#1– no known lung disease; Do#3&8– emphysema; Do7– scleroderma. *, *p*<0.05 *vs.* CTRL. Error bars, S.E.M.

Next, we examined TGF-β1 effects on CFTR mRNA levels. HBE cells were treated with TGF-β1 (15 ng/ml) added to the basolateral medium for 6, 12 or 24 h and the CFTR mRNA was measured by qRT-PCR. Despite differences in the fold change, TGF-β1 consistently decreased the CFTR mRNA in HBE cells from all donors (Fig. 3). These data demonstrate that TGF-β1 inhibits CFTR biogenesis by inhibiting CFTR mRNA levels in HBE cells.

### TGF-β1 Attenuates CFTR Mediated Cl^−^ Secretion in HBE Cells

**Figure 4:**
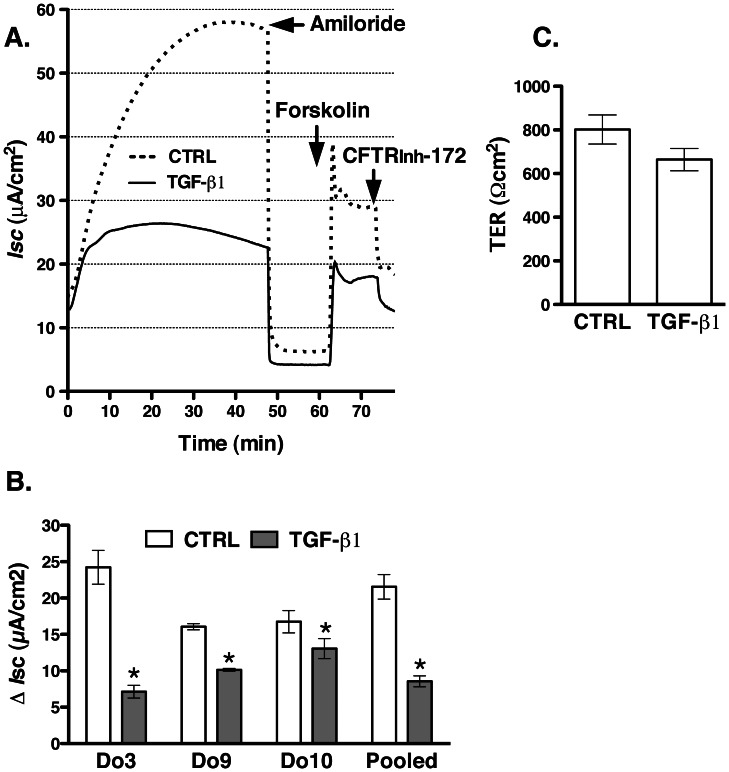
Ussing chamber experiments demonstrating that TGF-β1 inhibits CFTR mediated Cl^−^ secretion across HBE monolayers. TGF-β1 (15 ng/ml) or vehicle control (CTRL) was added to the basolateral medium and cells were incubated for 24 h. Subsequently, monolayers were mounted in Ussing chambers and bathed in Ringer’s solution. Amiloride (10 µM) was added to the apical bath solution to inhibit Na^+^ absorption through ENaC. *Isc* was stimulated with forskolin (10 µM) added to the apical and basolateral bath solution. Thiazolidonone CFTR inhibitor, CFTR_inh_-172 (20 µM) was added to the apical bath solution. Data are expressed as the CFTR_inh_-172 sensitive short-circuit current (*I*sc) calculated by subtracting the *Isc* after CFTR_inh_-172 treatment from the peak forskolin-stimulated *Isc*. Representative recordings (**A**) and summary of data (**B**&**C**) demonstrating that TGF-β1 decreased the CFTR_inh_-172 sensitive *I*sc in HBE cell from all examined donors without significantly changing the transepithelial resistance (TER; pooled data). As previously demonstrated TGF-β1 also attenuated the amiloride sensitive *I*sc [53]. 29 monolayers in the CTRL group and 33 monolayers in the TGF-β1 group were obtained from three donors (Do). The underlying diagnoses were: Do#3&9– emphysema; Do#10– bronchiolitis obliterans. *, *p*<0.05 *vs.* CTRL. Error bars, S.E.M.

Control of the CFTR mediated Cl^−^ secretion across epithelial cell monolayers is achieved at the level of both CFTR Cl^−^ channel activity and the plasma membrane protein abundance [41], [42]. Because inhibiting CFTR biogenesis by TGF-β1 decreased CFTR abundance at plasma membrane (Fig. 2D&E), we predicted that it would also inhibit CFTR mediated Cl^−^ secretion. HBE cells were treated with TGF-β1 (15 ng/ml) added to the basolateral medium for 24 h. TGF-β1 decreased the CFTR_inh_-172 sensitive short circuit current (*I*sc) across HBE monolayers though the magnitude of inhibition differed between HBE cells from different donors (Fig. 4). These data demonstrate that in HBE cells decreased biogenesis and depletion of CFTR from the plasma membrane by TGF-β1 inhibits CFTR mediated *I*sc. TGF-β1 did not significantly reduce the transepithelial resistance (TER) in HBE cells indicating that TGF-β1 did not compromise the integrity of HBE monolayers (Fig. 4C). These results are consistent with our biochemical data demonstrating integrity of HBE cells during TGF-β1 treatment because ezrin, an intracellular protein was not detected in the biotinylated (i.e. plasma membrane) samples (Fig. 2D).

**Figure 5:**
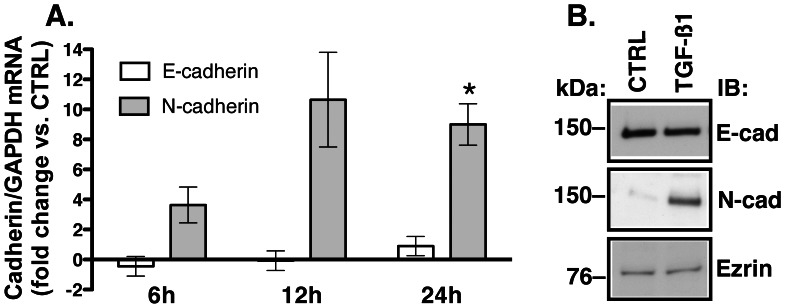
qRT-PCR and western blot experiments examining effects of TGF-β1 on the epithelial phenotype in HBE cells. To examine whether the 24 h treatment with TGF-β1 alters the epithelial phenotype of HBE cells we examined the mRNA levels (**A**) and protein abundance (**B**) of E- and N-cadherin, markers of epithelial and mesenchymal phenotype, respectively. TGF-β1 (15 ng/ml) or vehicle control (CTRL) was added to the basolateral medium for 6, 12, or 24 h. (**A**) qRT-PCR experiments. Raw data were analyzed using the ΔΔC_t_ method. Changes in the E- and N-cadherin mRNA were normalized to GAPDH. Data are expressed as fold change in E- or N-cadherin mRNA *vs.* CTRL. The 24 h treatment with TGF-β1 increased levels of N-cadherin mRNA without reducing the E-cadherin mRNA. All experiments were performed in triplicate in cells obtained from 3 donors. *, *p*<0.05 *vs.* CTRL. Error bars, S.E.M. (**B**) Western blot experiments demonstrating abundance of E-and N-cadherin in WCL after the 24 h treatment TGF-β1 or CTRL. Ezrin was used as a loading control. Experiment was repeated 3 times in HBE cells from different donors with similar results.

TGF-β1 induces a phenotype switch from polarized epithelial to motile mesenchymal called epithelial-to-mesenchymal transformation (EMT) [43]. The time required for the phenotype switch differs between cell types and may depend on the initial state of epithelial cell polarization and differentiation. To determine the temporal relationship between the TGF-β1 mediated inhibition of CFTR and EMT we examined the mRNA levels and protein abundance of E- and N-cadherin, markers of the epithelial and mesenchymal cell phenotype, respectively [44]. While TGF-β1 increased N-cadherin mRNA and protein levels it did not inhibit the biogenesis of E-cadherin (Fig. 5). Because a switch from E- to N-cadherin is the hallmark of EMT [45], these data indicate that while the EMT signaling has been initiated by TGF-β1 the phenotype switch has not occurred in 24 h. Together with biochemical and functional data confirming integrity of HBE monolayers, these results demonstrate that TGF-β1 inhibits CFTR biogenesis in HBE cells while the cells maintain epithelial phenotype.

### TGF-β1 Inhibits ΔF508-CFTR Biogenesis and Interferes with the Functional Rescue the ΔF508-CFTR

**Figure 6:**
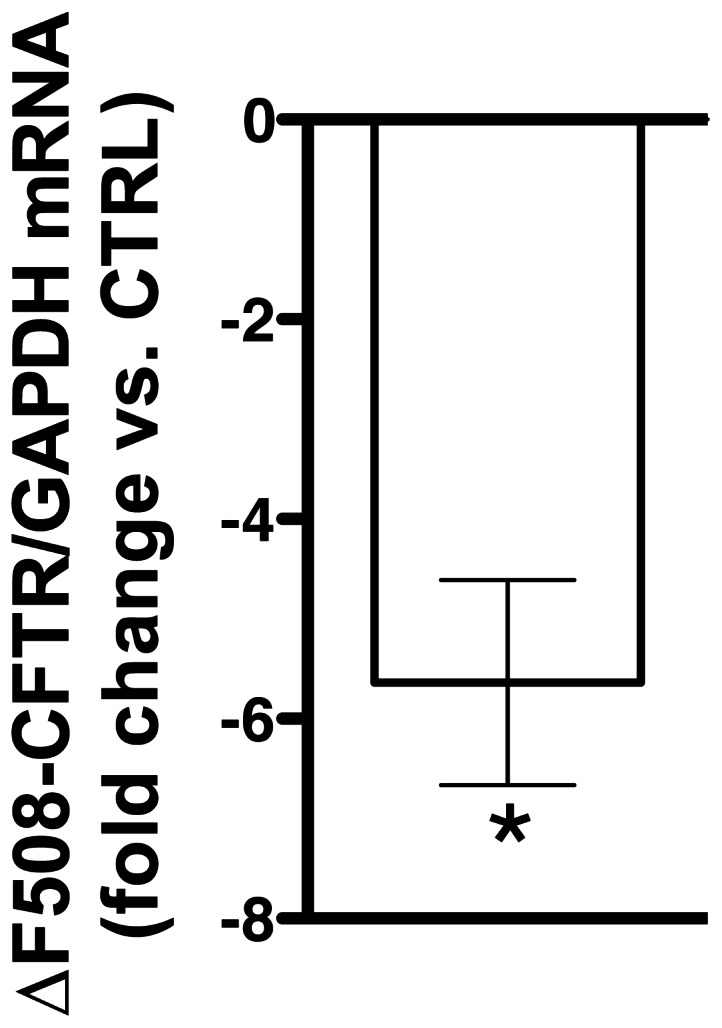
Summary of qRT-PCR experiments demonstrating that TGF-β1 decreases ΔF508-CFTR mRNA levels in CF-HBE cells. TGF-β1 (15 ng/ml) or vehicle control (CTRL) was added to the basolateral medium and cells were incubated for 24 h. Raw data were analyzed using the ΔΔC_t_ method. Changes in the ΔF508-CFTR mRNA were normalized to GAPDH. Data are expressed as fold change in ΔF508-CFTR mRNA *vs.* CTRL. All experiments were performed in triplicates in cell obtained from 3 donors. *, *p*<0.05 *vs.* CTRL. Error bars, S.E.M.

To address directly the effects of TGF-β1 on ΔF508-CFTR we studied cells from patients homozygous for the ΔF508 mutation (CF-HBE). TGF-β1 (15 ng/ml) added to the basolateral medium of CF-HBE monolayers for 24 h decreased the ΔF508-CFTR mRNA levels (Fig. 6).

**Figure 7:**
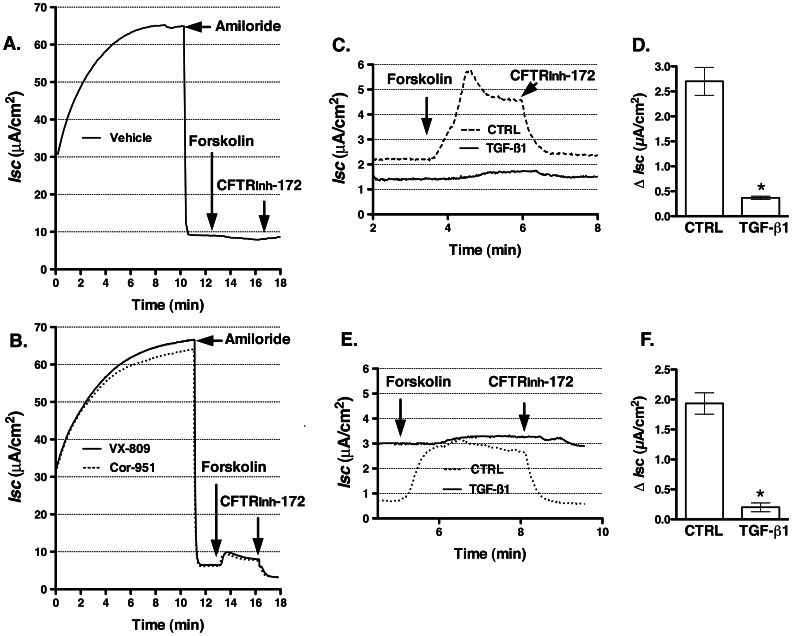
Using chamber experiments demonstrating that TGF-β1 inhibits functional rescue of ΔF508-CFTR in CF-HBE cells. Representative recordings (**A&B**) demonstrating that VX-809 and CF-106951 partially rescued the CFTR_inh_-172 sensitive *I*sc compared to vehicle control (Vehicle). VX-809 (10 µM), CF-106951 (10 µM) or vehicle control (DMSO) was added to the basolateral medium for 24 h. The final concentration of DMSO was <0.1%. Experiments were repeated at least 3 times in CF-HBE cells from 3 different donors. In subsequent experiments, corrector VX-809 (**C&D**) or CF-106951 (**E&F**) was used for 24 h to rescue the CFTR_inh_-172 sensitive *I*sc. Subsequently, TGF-β1 (15 ng/ml) or vehicle control (CTRL) was added with fresh VX-809 or CF-106951 to the basolateral medium for 24 h. Monolayers were bathed in Ringer’s solution in the presence of amiloride (10 µM). TGF-β1 decreased the CFTR_inh_-172 sensitive *I*sc rescued by either VX-809 or CF-106951. 6 monolayers/group from two CF-HBE cell donors (B–D). *, *p*<0.05 *vs.* CTRL. Error bars, S.E.M.

ΔF508-CFTR rescued by VX-809 is short-lived compared to wild-type CFTR [38]. Thus, factors that inhibit ΔF508-CFTR biogenesis would be expected to compromise the VX-809 mediated functional rescue of ΔF508-CFTR. Studies were conducted to examine TGF-β1 effects on the functional rescue of ΔF508-CFTR by two CFTR correctors, VX-809 and CF-106951. As demonstrated in Figure 7A&B, both correctors partially rescued the ΔF508-CFTR mediated *I*sc in CF-HBE cells at 24 h. The functional rescue of ΔF508-CFTR by either corrector was insufficient to detect rescue of ΔF508-CFTR at the protein level (data not shown). Subsequently, cells treated for 24 h with corrector VX-809 or CF-106951 were incubated with either TGF-β1 or vehicle control in the presence of fresh corrector for another 24 h. TGF-β1 inhibited the ΔF508-CFTR mediated *I*sc rescued by either VX-809 or CF-106951 (Fig. 7C–F). These data show that in primary differentiated human bronchial epithelial cells TGF-β1 interferes with the functional rescue of ΔF508-CFTR. The TGF-β1 effects were independent of the mechanisms or sites of action of CFTR correctors because TGF-β1 inhibited the functional rescue of ΔF508-CFTR achieved by both correctors. Taken together the above results suggest that activation of TGF-β1 signaling may compromise rescue of defective biosynthetic processing of ΔF508-CFTR by the CFTR correctors.

## Discussion

The major novel observation in the present study is that TGF-β1 inhibits CFTR biogenesis by decreasing CFTR mRNA levels and protein abundance in primary differentiated human bronchial epithelial cells from non-CF individuals and from patients homozygous for the ΔF508 mutation. Moreover, TGF-β1 inhibits CFTR mediated *I*sc and compromises the efficacy of CFTR correctors by inhibiting the functional rescue of ΔF508-CFTR.

Several lines of evidence in the present study support these conclusions. Compared to controls, treatment of HBE cells with clinically relevant TGF-β1 concentrations [23], [40], [46] activated the canonical TGF-β pathway, inhibited CFTR mRNA levels, and decreased CFTR protein abundance in a time and concentration dependent manner (Figs. 1–3). Depletion of CFTR from WCL correlated with decreased CFTR abundance in the plasma membrane and with the reduction of CFTR mediated *I*sc (Figs. 2D&4). TGF-β1 inhibited CFTR biogenesis without compromising the epithelial phenotype or integrity of HBE cells (Figs. 2D, 4C&5). Moreover, TGF-β1 decreased the ΔF508-CFTR mRNA levels and inhibited the corrector mediated functional rescue of ΔF508-CFTR in CF-HBE cells (Fig. 6&7).

Published studies have demonstrated that TGF-β1 affects CFTR expression and function in some epithelial cell models, although none of these studies has established the effects of TGF-β1 on CFTR in primary differentiated human bronchial epithelial cells from non-CF individuals or CF patients. First, TGF-β1 inhibits CFTR and regulates water balance in human colonic epithelial cells [26]. Second, TGF-β1 inhibits CFTR in epithelial cells from non-CF patients with nasal polyps [27]. The clinical significance of this finding remains unclear because non-CF patients with nasal polyps demonstrate decreased TGF-β1 levels compared to controls [22]. Third, TGF-β1 inhibits CFTR expression and function in rat alveolar epithelial cells [28].

By contrast, our study demonstrates that in primary differentiated human bronchial epithelial cells TGF-β1 inhibits CFTR expression and function by an EMT independent mechanism. TGF-β1 is an important mediator of EMT and plays an integral role in repair and scar formation following epithelial injury and contributes to development of fibrosis [47]. Our data indicate that factors known to activate TGF-β1 signaling, such as infections, environmental toxins, tobacco smoke exposure [40], [48], [49] could inhibit CFTR biogenesis *in vivo* even prior to an established airway epithelial cell injury. We do not know why exogenous TGF-β1 elicited such a wide range of CFTR inhibition in HBE cells from different donors (Figs. 2C, 3A & 4B). Unlike immortalized and clonally selected cells, primary differentiated cells, including HBE retain many features of the unique *in vivo* cellular environment. Thus, differences in the magnitude of TGF-β1 mediated CFTR inhibition in our study could result from the cell donor-specific differences in the endogenous activity of TGF-β pathway, *TGF-β* gene polymorphisms, other cytokines affecting CFTR, and additional epigenetic factors [14]–[18].

There are no published data on how TGF-β1 inhibits CFTR mRNA level or whether a putative TGF-β1 consensus site exists in the CFTR promoter. We do not know whether TGF-β1 inhibits CFTR expression by transcriptional or postransctiprional mechanisms in HBE cells. Studies designed to address these questions are in progress. The complexity and versatility of the TGF-β pathway indicate that several mechanisms, including direct and indirect may play a role in modulating CFTR expression.

Our study demonstrates that TGF-β1 may inhibit the corrector mediated functional rescue of ΔF508-CFTR. In the CF airway, TGF-β1 signaling is activated by *Pseudomonas aeruginosa*, poor nutritional status, or by certain *TGF-β* gene polymorphisms [40], [46], [50]–[52]. According to our results, increased TGF-β1 signaling could explain at least in part the disappointing effects of the investigational drug VX-809 in a recent clinical trial [10]. Future studies are needed to examine whether TGF-β1 or other biomarkers of the CF lung disease could assist in predicting efficacy of therapies designed to correct the ΔF508-CFTR processing defect. Such biomarkers may serve to rigorously evaluate investigational drugs *in vitro* and may help to individualize future disease-modifying approaches in CF patients.
